# Erratum for Westmeier et al., “Impaired Cytotoxic CD8^+^ T Cell Response in Elderly COVID-19 Patients”

**DOI:** 10.1128/mBio.02805-20

**Published:** 2020-11-10

**Authors:** Jaana Westmeier, Krystallenia Paniskaki, Zehra Karaköse, Tanja Werner, Kathrin Sutter, Sebastian Dolff, Marvin Overbeck, Andreas Limmer, Jia Liu, Xin Zheng, Thorsten Brenner, Marc M. Berger, Oliver Witzke, Mirko Trilling, Mengji Lu, Dongliang Yang, Nina Babel, Timm Westhoff, Ulf Dittmer, Gennadiy Zelinskyy

**Affiliations:** a Institute for Virology, University Hospital Essen, University of Duisburg-Essen, Essen, Germany; b Center for Translational Medicine, Medical Department I, Marien Hospital Herne, University Hospital of the Ruhr–University Bochum, Herne, Germany; c Department of Infectious Diseases, West German Centre of Infectious Diseases, University Hospital Essen, University Duisburg-Essen, Essen, Germany; d Department of Anesthesiology, University Hospital Essen, University Duisburg-Essen, Essen, Germany; e Department of Infectious Diseases, Union Hospital of Tonji Medical College, Huazhong University of Science and Technology, Wuhan, China; f Joint International Laboratory of Infection and Immunity, HUST, Wuhan, China; g Charité–Universitätsmedizin Berlin, Corporate Member of Freie Universität Berlin, Humboldt-Universität zu Berlin, Berlin, Germany; h Berlin Institute of Health, Berlin-Brandenburg Center for Regenerative Therapies, Berlin, Germany; i Medical Department I, Marien Hospital Herne, University Hospital of the Ruhr University of Bochum, Bochum, Germany

## ERRATUM

Volume 11, no. 5, e02243-20, 2020, https://doi.org/10.1128/mBio.02243-20. The affiliation for Dr. Paniskaki was shown wrongly. The affiliation should appear as shown above. The article has been updated online.

