# Supplementary material for: Impaired Cytotoxic CD8+ T Cell Response in Elderly COVID-19 Patients
Source: mBio. 2020 Sep 18;11(5):e02243-20. doi: 10.1128/mBio.02243-20 (PMC7502863; doi:10.1128/mBio.02243-20)
Supplement: TABLE S1 [file mBio.02243-20-st001.docx]

**Supplement Table 1. Laboratory Characteristics of Patients according to Age**

|  | Age (years) | |  |
| --- | --- | --- | --- |
|  | 29-79 (median 62) | 80-96 (median 86) |  |
| Number of Patients | 22 (7F, 15M) | 8 (3F, 5M) |  |
|  |  |  |  |
|  |  |  |  |
| Median laboratory findings and range |  |  |  |
|  |  |  |  |
| White cell count-per µl | 6900(4400-10800) | 8200(4500-18000) |  |
| Red cell count-per pl | 3.7(2.2-4.5) | 4.1(3.7-4.8) |  |
| Lymphocyte count-per µl | 920(390-1500) | 790(410-1400) |  |
| Neutrophile count-per µl | 5200(900-8700) | 6400(2500-10700) |  |
| Platelet count-per nl | 218(142-390) | 211(112-383) |  |
| Monocytes count-per µl | 440(250-950) | 540(200-1030) |  |
| Hemoglobin-g/dl | 11.3(7.9-15.6) | 10.7(7.2-16.7) |  |
| Haematocrit l/l | 0.332(0.26-0.39) | 0.315 (0.176-0.420) |  |
| C-reactive protein - mg/dl | 9.4(1.3-17.5) | 6.8(2-9.4) |  |
| Procalcitonin - ng/ml | 0.11(0.02-0.68) | 0.12(0.01-1.48) |  |
| Lactat dehydrogenase - U/L | 337(187-610) | 302(184-655) |  |
| Aspartate aminotransferase - U/L | 34(11-115) | 37(13-123) |  |
| Alanine aminotranserase - U/L | 24(10-121) | 32(13-136) |  |
| Gamma-glutamyl transferase  - U/L | 80.6(22-290) | 85.8(14-281) |  |
| Total Bilirubin - mg/dl | 0.8(0.2-1.8) | 0.6(2.2-1.4) |  |
|  |  |  |  |
|  |  |  |  |
|  |  |  |  |
