# Supplementary figures and images for: Impaired Cytotoxic CD8+ T Cell Response in Elderly COVID-19 Patients
Source: mBio. 2020 Sep 18;11(5):e02243-20. doi: 10.1128/mBio.02243-20 (PMC7502863; doi:10.1128/mBio.02243-20)

# Supplement Fig. S1

Lymphocytes

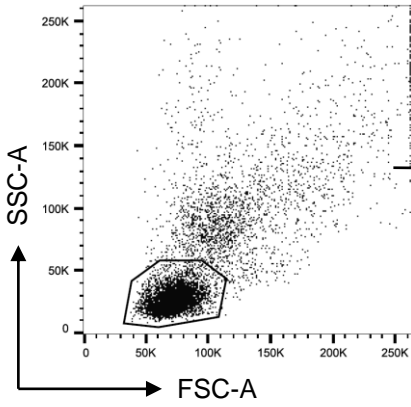

Single cells

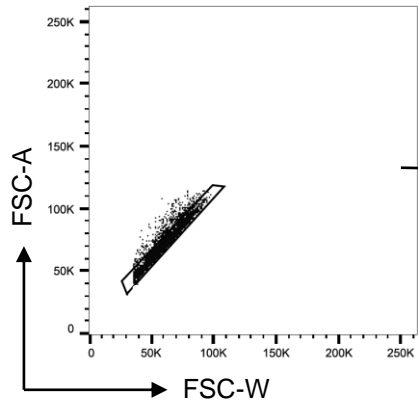

Viable CD3<sup>+</sup>

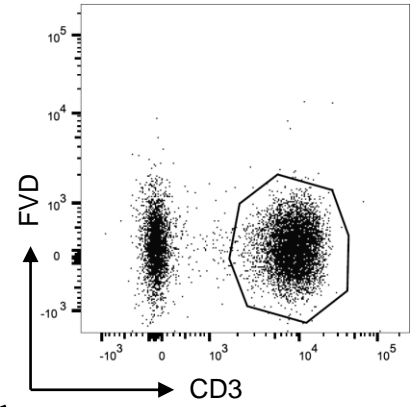

CD4<sup>+</sup> T cells

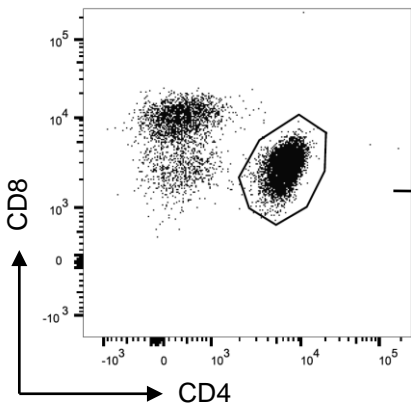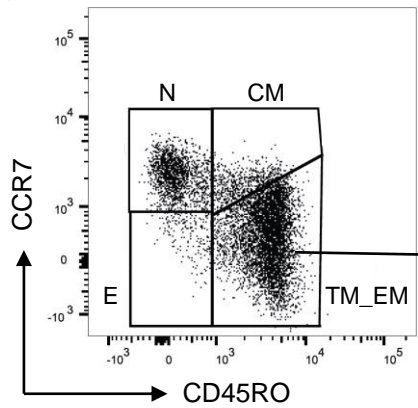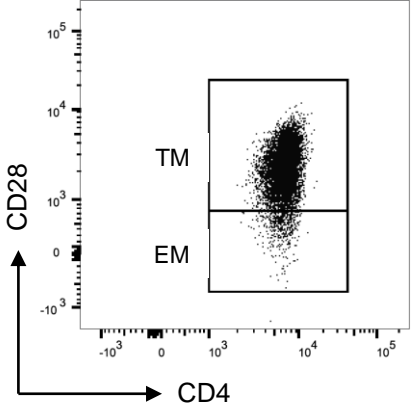

Supplement: FIG S1 [file mBio.02243-20-sf001.pdf]

## Supplement Fig. S2

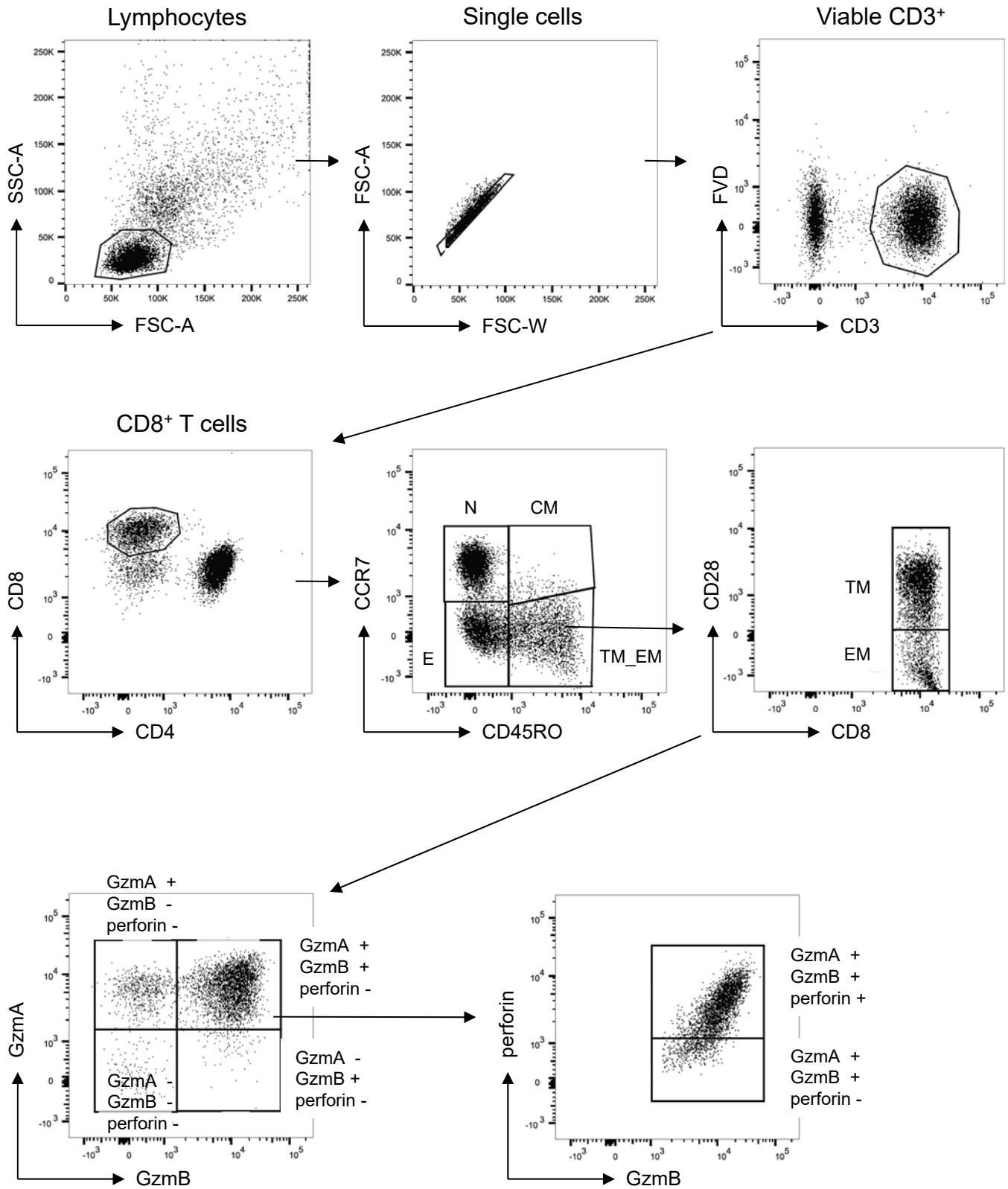

Supplement: FIG S2 [file mBio.02243-20-sf002.pdf]

Supplement Fig. S3

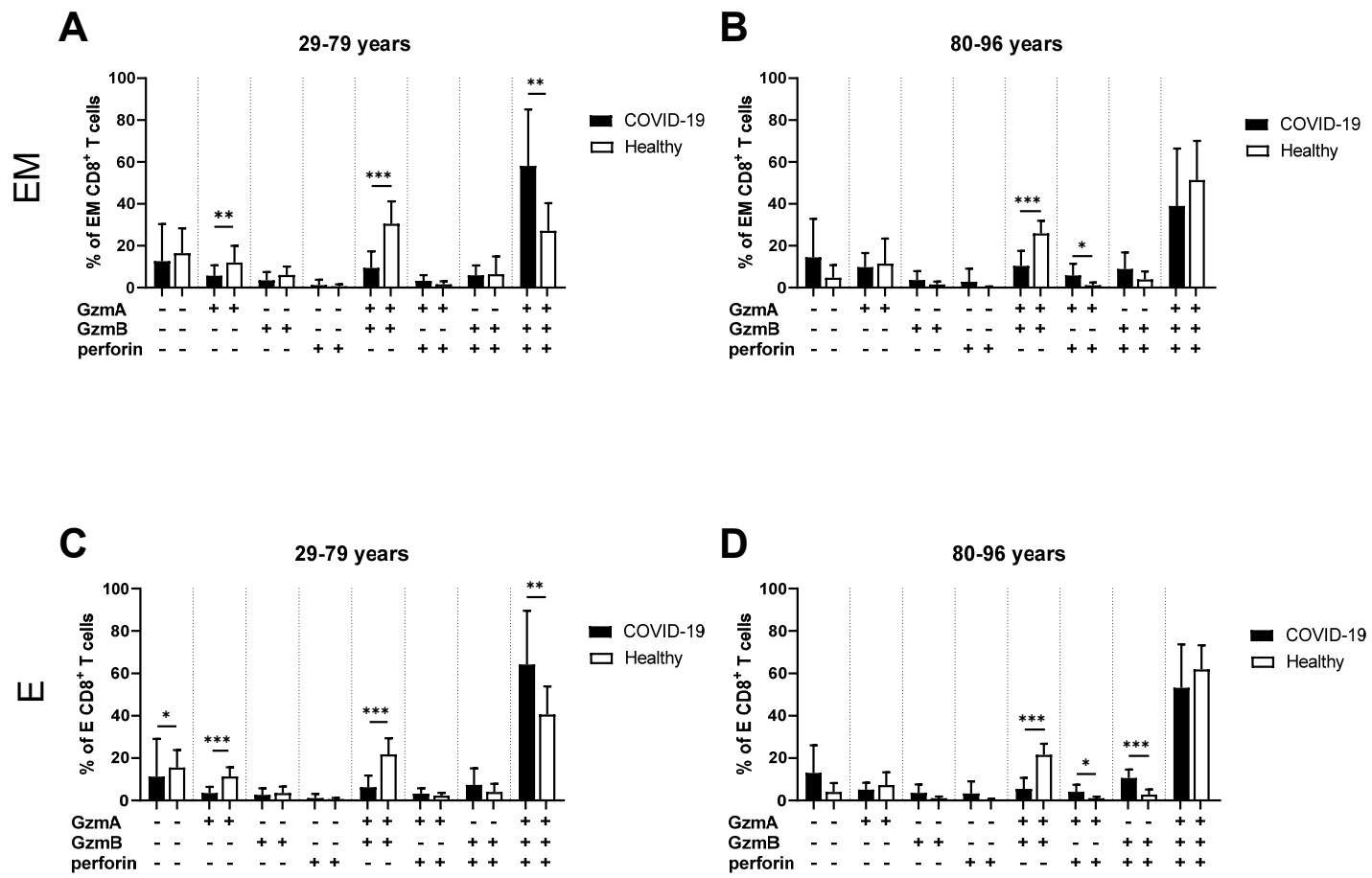

Supplement: FIG S3 [file mBio.02243-20-sf003.pdf]
